# Quantitative proteomic analysis by iTRAQ^® ^for the identification of candidate biomarkers in ovarian cancer serum

**DOI:** 10.1186/1477-5956-8-31

**Published:** 2010-06-14

**Authors:** Kristin LM Boylan, John D Andersen, Lorraine B Anderson, LeeAnn Higgins, Amy PN Skubitz

**Affiliations:** 1 Department of Laboratory Medicine and Pathology, University of Minnesota, MMC 609, 420 Delaware St. SE, Minneapolis, MN, USA; 2 Department of Biochemistry, Molecular Biology, and Biophysics, University of Minnesota, 6-155 Jackson Hall, 321 Church St SE, Minneapolis, MN, USA

## Abstract

**Background:**

Ovarian cancer is the most lethal gynecologic malignancy, with the majority of cases diagnosed at an advanced stage when treatments are less successful. Novel serum protein markers are needed to detect ovarian cancer in its earliest stage; when detected early, survival rates are over 90%. The identification of new serum biomarkers is hindered by the presence of a small number of highly abundant proteins that comprise approximately 95% of serum total protein. In this study, we used pooled serum depleted of the most highly abundant proteins to reduce the dynamic range of proteins, and thereby enhance the identification of serum biomarkers using the quantitative proteomic method iTRAQ^®^.

**Results:**

Medium and low abundance proteins from 6 serum pools of 10 patients each from women with serous ovarian carcinoma, and 6 non-cancer control pools were labeled with isobaric tags using iTRAQ^® ^to determine the relative abundance of serum proteins identified by MS. A total of 220 unique proteins were identified and fourteen proteins were elevated in ovarian cancer compared to control serum pools, including several novel candidate ovarian cancer biomarkers: extracellular matrix protein-1, leucine-rich alpha-2 glycoprotein-1, lipopolysaccharide binding protein-1, and proteoglycan-4. Western immunoblotting validated the relative increases in serum protein levels for several of the proteins identified.

**Conclusions:**

This study provides the first analysis of immunodepleted serum in combination with iTRAQ^® ^to measure relative protein expression in ovarian cancer patients for the pursuit of serum biomarkers. Several candidate biomarkers were identified which warrant further development.

## Background

Ovarian cancer results in over 14,000 deaths each year, making it the fifth leading cause of cancer-related deaths for women in the United States [1]. The high mortality rate is due, in part, to the fact that over 80% of cases are diagnosed after the cancer has spread beyond the ovary. When ovarian cancer is detected early, the survival rate is over 90% [2], highlighting the need for biomarkers for early detection.

Current biomarkers for ovarian cancer detection and screening are inadequate. The antigen CA125 is elevated in the sera of most patients diagnosed with ovarian cancer [3,4]. However, CA125 lacks the sensitivity and specificity required for general screening, although it is commonly used to monitor for recurrence. Many researchers have attempted to find protein biomarkers for ovarian cancer to replace or be used in conjunction with CA125 in order to improve the sensitivity and specificity of diagnostic tests (reviewed in [5]).

Recently, methods for quantitative MS-based proteomics have allowed the direct comparison of protein levels present in control and diseased samples. Two technologies for quantitative proteomic studies are ICAT^® ^[6] and iTRAQ^® ^[7], which employ differential labeling of up to eight protein samples using stable isotope "chemical tags," and analysis by mass spectrometry. Stewart et al. [8] have used ICAT^® ^to quantify differences between a cisplatin-resistant and a cisplatin-sensitive ovarian cancer cell line. Gagne et al. [9] used iTRAQ^® ^and 2-DE to evaluate differential protein expression between an ovarian cancer cell line of low malignant potential with a highly proliferative cell line. Whether any of the proteins identified in these studies are present in patients' sera is not known.

The MS identification of tumor-derived proteins in plasma is hampered by the presence of a few highly abundant proteins, which can mask the detection of low abundance proteins which may be used as biomarkers. We recently reported the depletion of high abundance proteins from pooled serum samples from 60 patients with serous ovarian carcinoma and 60 non-cancer controls using immunoaffinity depletion columns. The remaining medium and low abundance proteins were then subjected to analysis by DIGE in order to identify proteins with increased abundance in ovarian cancer sera relative to control sera that may represent specific biomarkers [10]. In this study, we used iTRAQ^® ^labeling to quantitate the medium and low abundance proteins in serum for the identification of candidate ovarian cancer biomarkers. This analysis is the first to use immunodepletion in combination with iTRAQ^® ^labeling to look directly in sera for ovarian cancer-specific biomarkers.

## Results

### Identification of candidate biomarkers by iTRAQ^®^

**Figure 1:**
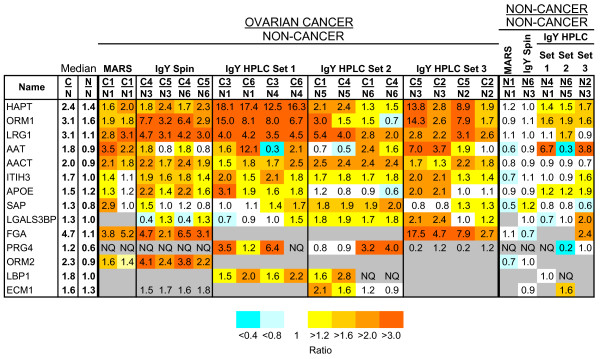
**iTRAQ^® ^Ratios for Selected Proteins Identified in Ovarian Cancer and Non-cancer Serum Pools**. iTRAQ^® ^ratios from all experiments for proteins that met our criteria for upregulation in at least one experiment (i.e. the ratio of cancer/non-cancer (C/N) was 1.6 or more and the corresponding non-cancer/non-cancer (N/N) ratio was between 0.8 and 1.2). NQ, Not quantified. Gray boxes, not detected or quantified with 1 low confidence peptide. The p-value, EF and number of peptides for each protein, as well as the accession number, are listed in Additional Files Table 1. HAP, haptoglobin; ORM1, orosomucoid 1; LRG, leucine rich alpha-2 glycoprotein 1; AAT, alpha-1 antitrypsin; AACT, alpha-1 antichymotrypsin; ITIH3, inter-alpha globulin inhibitor H3; APOE, apolipoprotein E; SAP, serum amyloid P component; LGALS3BP, galectin-3 binding protein; FGA, fibrinogen-alpha; PRG4, proteoglycan 4; ORM2, orosomucoid 2; LBP1, lipopolysaccharide binding protein 1; ECM1, extracellular matrix protein 1.

To reduce the dynamic range of proteins in the serum and increase the likelihood of identifying medium and low abundance serum proteins by mass spectrometry, we used three commercial immunoaffinity depletion methods prior to proteomic analysis: the multiple affinity removal system (MARS) column, the IgY-12 spin cartridge, or the IgY-12 HPLC column [10]. Serum samples from 60 patients with serous ovarian carcinoma and 60 non-cancer controls were randomly combined into six pools containing 10 patient samples each (e.g. C1 to C6 for the 60 cancer samples; N1 to N6 for the 60 non-cancer control samples). In order to identify proteins that are differentially expressed in ovarian cancer serum relative to control, pooled, depleted sera were labeled with isobaric tags using iTRAQ^® ^for the relative quantitation of the medium and low abundance proteins. A summary of the five iTRAQ^® ^experiments is shown in Table 1. Protein levels were considered increased in sera from ovarian cancer patients relative to non-cancer controls if the iTRAQ^® ^ratio of cancer/non-cancer (C/N) was ≥ 1.6 and the corresponding non-cancer/non-cancer (N/N) ratio was between 0.8 and 1.2. Fourteen proteins met these criteria for overexpression in one or more experiments. The relative expression of these proteins for each serum pool is displayed in Figure 1. The relative expression, statistical parameters for quantitation, and the peptide information for these proteins are provided in Additional files 1 and 2, Tables S1 and S2.

**Table 1:** Summary of the number of proteins identified in serum after abundant protein depletion

	**iTRAQ^® ^Mass Tag**				**Number of Proteins Identified** **Confidence Level**		**Proteins Increased > 1.6 Fold**^†^
**Depletion Experiment**	**114**	**115**	**116**	**117**	**≥ 99%**	**≥ 95%**	
		
MARS	nd	C1	N1	N1	75	86	5
IgY-12 Spin column	C4	N3	C5	N6	114	138	9
IgY-12 HPLC set 1	N1	N4	C3	C6	88	107	5
IgY-12 HPLC set 2	N5	N6	C1	C4	109	134	4
IgY-12 HPLC set 3	N3	N2	C5	C2	87	113	2

C1-C6 correspond to six pools of ovarian cancer serum (10 patients each).

N1-N6 correspond to six pools of non-cancer control serum (10 patients each).

nd, not done

^†^C/N ratio > 1.6 and N/N ratio between 0.8 and 1.2

### iTRAQ^® ^analysis of sera depleted by MARS

The medium and low abundance proteins from ovarian cancer pool C1 and non-cancer pool N1 depleted using the MARS column contained a total of 86 proteins identified with ≥ 95% confidence (Table 1). The five proteins that met our criteria for an increase in relative abundance in the cancer compared to the control were haptoglobin (HAPT), orosomucoid-1 (ORM1), leucine-rich alpha-2-glycoprotein-1 (LRG1), alpha-1 antichymotrypsin (AACT), and fibrinogen-alpha (FGA; Figure 1; Additional file 1, Table S1). Several proteins that were identified in the MARS iTRAQ^® ^experiment by single peptide hits were identified by at least two peptides, with significantly increased ratios in the other iTRAQ^® ^analyses (Figure 1; Additional file 1, Table S1), including: LRG1, inter-alpha globulin inhibitor-H3 (ITIH3), and proteoglycan-4 (PRG4). Although identified based on a single peptide, the average iTRAQ^® ^protein ratio for LRG1 was made from three (replicate) peptide spectra, and the ratio was ~2.9-fold higher in ovarian cancer relative to control sera. The iTRAQ^® ^protein ratio for inter-alpha globulin inhibitor-H3 was based on a single peptide, while no iTRAQ^® ^ratio was reported for PRG4 (Figure 1).

### iTRAQ^® ^analysis of sera depleted by the IgY12 spin column

Four serum pools (C4, C5, N3, and N6) depleted with the IgY12 spin column were labeled with iTRAQ^® ^mass tags and analyzed by MS (Table 1). Of the 138 proteins that were identified at ≥ 95% confidence, nine were overexpressed in the ovarian cancer compared to the control serum pools (Table 1). Four of the five proteins that were elevated in the MARS experiment were also increased in this experiment. In addition, five new proteins with increased expression in ovarian cancer sera were identified: alpha-1-antitrypsin (AAT), apolipoprotein E (APOE), extracellular matrix protein-1 (ECM1), inter-alpha globulin inhibitor-H3, orosomucoid-2 (ORM2; Figure 1; Additional file 1, Table S1). Orosomucoid-2 was identified in the MARS depletion experiment, but did not meet our criteria for increased relative expression in ovarian cancer sera. Two proteins (serum amyloid P-component (SAP) and ECM1) were identified based on single peptide hits in this experiment, but were identified by two or more peptides in at least one other experiment (Additional files 1 and 2, Tables S1 and S2).

### iTRAQ^® ^analysis of sera depleted by the IgY12 HPLC

iTRAQ^® ^analyses were performed on sera depleted by the IgY12 HPLC column for all six pairs of cancer and control serum pools, in three separate experiments (Table 1). The maximum number of proteins identified at a ≥ 95% confidence level was 134 (HPLC set 2). The number of proteins that were overexpressed in the cancer compared to the control serum pools ranged from two proteins (HPLC set 3) to five proteins (HPLC set 2). Fibrinogen-alpha was identified only in set 3, while lipopolysaccharide binding protein-1 (LBP1) was identified only in sets 1 and 2, based on a single peptide in each experiment (Figure 1; Additional file 1, Table S1; spectra shown in Additional file 3, Figure S1). In addition to LBP1, the overexpressed proteins identified in the three IgY12 HPLC experiments included four proteins identified by single peptides, however with the exception of LBP1, these were all identified by 2 or more peptides in the other experiments (Additional file 1, Table S1). Galectin-3-binding protein (LGALS3BP) was identified in each IgY12 HPLC experiment; however, it met our criteria for increased expression only in HPLC set 2.

### Comparison of Proteins Identified in Immunodepleted Sera

**Figure 2:**
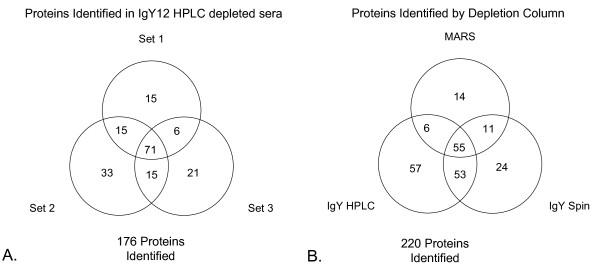
**Proteins identified in immunodepleted sera**. Venn diagrams showing the number of total proteins identified by MS/MS (≥ 95% confidence) in immunodepleted sera. (A) Proteins identified in the three sets of sera depleted by the IgY12 HPLC column. (B) Proteins identified in sera depleted by the MARS, IgY12 spin, and IgY12 HPLC columns. Identified proteins are listed in Additional file 2, Table S2.

Proteins identified by MS/MS were compared among the three immunodepletion methods. Figure 2A compares the proteins identified in the three sets of experiments run on IgY12 HPLC depleted sera. Of the 176 total proteins identified, 40% of the proteins were identified in all three experiments; and between 8 and 19% of the total were unique to a single experiment.

Comparing the different immunoaffinity columns (Figure 2B) demonstrates that 25% of the 220 proteins were identified by all three immunoaffinity depletion methods. Not surprisingly, the proteins identified in serum depleted by the two IgY12 column formats were more similar to each other (i.e. 50% shared proteins) than to the MARS column. About 40% more total proteins were identified in the IgY12-depleted sera than in the MARS-depleted sera; however, more immunoglobulins and complement proteins were identified in IgY12-depleted sera than in MARS-depleted sera (Additional file 2, Table S2). Taken together, these data suggest that depletion of the twelve most highly abundant serum proteins using the IgY12 column is more effective at reducing the dynamic range of serum proteins than the MARS column, and results in the identification of more medium and low abundance serum proteins by MS. Overall, twice as many proteins with increased expression in ovarian cancer sera were identified in the IgY12-depleted sera than were identified in the MARS-depleted sera.

### Validation of protein expression by immunoblotting

**Figure 3:**
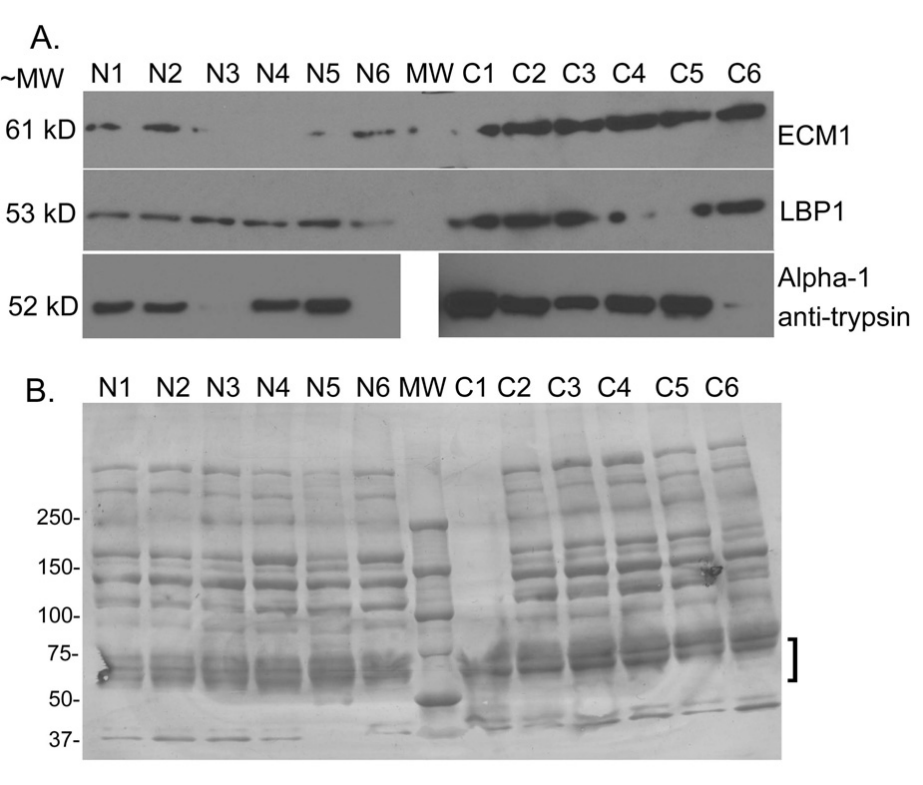
**Western blot validation of proteins overexpressed in cancer sera**. (A) Western blots of 50 μg of medium and low abundance proteins from each non-cancer (N1-N6) and cancer (C1-C6) serum pool depleted by the IgY12 HPLC column were probed with the antibodies to ECM1, LBP1, and alpha-1 antitrypsin. (B) A replicate of the PVDF blot was stained for total protein with colloidal gold as a control for loading/transfer. Bracket indicates the approximate region of the blot where proteins of interest would be expected.

Results of the iTRAQ^® ^experiments were validated by Western immunoblotting experiments (Figure 3). We have previously shown by DIGE analysis of immunodepleted sera that levels of AACT and LRG1 expression are increased in ovarian cancer sera, and validated this by Western blot [10]. Immunodepleted serum was used for the initial validation, as the presence of highly abundant serum proteins interferes with detection of medium and low abundance proteins by Western blot. Antibodies to ECM1, LBP1, and alpha-1 antitrypsin were used to probe Western blots of medium and low abundance proteins from serum pools depleted by the IgY12 HPLC column. We were particularly interested in validating the proteins for which the peptide identification and/or quantitation values were weak, as they represent proteins of very low abundance that may be tissue leakage proteins or proteins shed from the tumor. For example, ECM1 and LBP1 were identified in two of five iTRAQ^® ^experiments with 1 or 2 peptides (Additional file 1, Table S1). These proteins were both increased by 1.6-fold or greater in the iTRAQ^® ^experiments, and were correspondingly increased in ovarian cancer serum by Western blot (Figure 3). We also validated the level of serum expression for alpha-1 antitrypsin. By Western blot, alpha-1 antitrypsin, a protein of ~52 kDa, is markedly more intense in all of the cancer pools except C6. In an attempt to validate the iTRAQ^® ^quantitation for PRG4, two different commercial antibodies were used to probe Western blots of the depleted serum pools; however, the results of these blots were inconclusive, likely due to extensive post-translational modifications found in PRG4 [11].

## Discussion

We used the quantitative proteomic technique iTRAQ^® ^to analyze pooled, immunodepleted serum from ovarian cancer patients and non-cancer controls for the discovery of proteins that are present at increased levels in ovarian cancer serum and may be candidates for biomarkers. For our biomarker discovery experiments, we selected patients with advanced disease under the assumption that these patients may have higher levels of ovarian cancer proteins in their sera than patients with early stage disease, thus increasing the likelihood of detecting ovarian cancer specific biomarkers. The serum samples were pooled in order to reduce variation between individual samples and to increase the overall number of samples tested. The technique of pooling samples prior to proteomic analysis has been performed by others in similar studies with body fluids [12-14].

Relative expression levels of 14 proteins in serum from ovarian cancer patients were 1.6-fold or more higher than the corresponding serum protein levels of healthy women in at least one experiment. Nine proteins were identified in all five iTRAQ^® ^experiments, two proteins were identified in four experiments, two proteins were identified in three experiments, and one protein was identified in two experiments.

Several of the proteins identified in this study have previously been identified as potential ovarian cancer biomarkers [15-18], demonstrating that iTRAQ^® ^labeling of serum can be used for the identification of differentially expressed serum protein biomarkers. For example, haptoglobin was first shown to be elevated in the sera of women with ovarian cancer almost 40 years ago [16]. Others have confirmed this finding numerous times, most recently by 2DE [19], and also with SELDI and ELISA [17]. In addition to the iTRAQ^® ^data presented here, we have also shown by DIGE analysis that several haptoglobin subunits are present at elevated levels in ovarian cancer sera [10]. Serum amyloid A1 [20,21], and C-reactive protein [22,23] have also previously been shown to be elevated in ovarian cancer; in our analysis of iTRAQ^® ^labeled sera these proteins had average C/N ratios of 8.9 and 6.5 respectively, although the N/N peak height was below the threshold for quantitation and not reported by the ProteinPilot™ software. Consequently, these proteins did not meet our criteria for inclusion in Figure 1; however the relative expression, statistical parameters for quantitation, and the peptide information for these proteins are in Additional file 1, Table S1. C-reactive protein was identified by a single peptide in sera depleted by the MARS column; the spectrum for this peptide is shown in Additional file 3, Figure S1.

Notably, we identified several candidate biomarker proteins which have not been extensively studied. For example, apolipoprotein-E (ApoE) was identified by multiple peptides in all five iTRAQ^® ^experiments, and was ~1.6-fold elevated in the ovarian cancer serum pools. Although commonly known for its role in lipid transport, ApoE has previously been shown to be overexpressed in ovarian cancer cells [24,25] where it is required for proliferation and survival [25].

The protein LRG1 was determined to have robustly increased levels of expression in ovarian cancer sera by iTRAQ^®^. We have previously shown an increase in several LRG1 isoforms in ovarian cancer serum by DIGE [10]. LRG1 expression in ovarian cancer sera was validated by Western blot in the immunodepleted serum pools, and by ELISA in individual, undepleted serum samples (data not shown). Although LRG1 has been classified as an "acute phase" protein, produced by the liver in response to injury or infection [26,27], we have evidence that LRG1 is also being produced by ovarian cancer cells and may contribute to the increased levels of LRG1 found in patient sera (manuscript in preparation). LRG1 levels have also been shown to be elevated in the medium and low abundance serum proteins of lung and pancreatic cancers [28-30].

ECM1 was identified in two iTRAQ^® ^experiments, with an ovarian cancer-to-control ratio of ~1.6. The increase in expression of ECM1 was validated by Western blot in all of the ovarian cancer serum pools. ECM1 is a secreted protein, which has been shown to be overexpressed in a number of different epithelial cell tumors [31]. Interestingly, ECM1 was identified in two proteomic analyses of ovarian cancer ascites cells [32,33], and was also highly enriched in the conditioned media from ovarian cancer cell lines [33] and in chemoresistant ovarian tumor tissue [34].

PRG4 was identified by iTRAQ^®^, with a relative protein abundance in ovarian cancer-to-control of ~2.1. PRG4, also known as megakaryocyte stimulating factor and hemangiopoietin, is a multifunctional proteoglycan with growth promoting properties in hemangioblasts [35]. It is synthesized by chondrocytes and plays a role in joint lubrication [36], however it is also synthesized by the liver, heart, lung, and bone [37].

LBP1 was identified with an iTRAQ^® ^ratio of 1.95 in ovarian cancer vs. control sera, which was validated by Western blot. LBP1 is proposed to play a role in immune activation [38], however, the identification of LBP1 in ovarian cancer ascites cells using MS [32,33] also supports a potential role in ovarian cancer.

We have previously reported the identification of candidate serum biomarkers for ovarian cancer by DIGE analysis of pooled, depleted serum [10]. Four proteins were shown to have increased levels in ovarian cancer serum by both proteomic methods: haptoglobin, alpha-1 antichymotrypsin, serum amyloid P-component, and LRG1. Five other proteins were found at increased levels in ovarian cancer serum by DIGE, while 12 additional proteins were discovered by iTRAQ^®^, demonstrating that these two techniques for identification of candidate biomarkers are complementary.

Our study is the first to report immunoaffinity depletion in combination with iTRAQ^® ^labeling as a means to quantitate serum proteins in ovarian cancer. Gagne et al. [9] used the quantitative proteomic techniques of both iTRAQ^® ^and 2DE to compare two ovarian cancer cell lines, one highly proliferative and the other with low malignant potential, to identify proteins associated with invasion and metastasis. Similarly, proteins associated with prostate cancer progression were identified using iTRAQ^® ^labeling and comparison of highly and poorly metastatic prostate cancer cell lines [39]. Other iTRAQ^® ^studies have compared tumor to normal tissue for biomarker discovery (e.g. endometrial, head and neck cancers [40-42], and hepatocellular carcinoma [43]). iTRAQ^® ^analysis of biofluids such as saliva [14] and cerebral spinal fluid [12] has also been reported. Interestingly, a recent quantitative proteomic analysis of ovarian tumor tissue using ICAT labeling examined the relative expression of tumor proteins in chemoresistant vs. chemosensitive tumors, and found that five of the proteins identified in this study [ECM1, LRG1, orosomucoid 1 (alpha-1-acid glycoprotein) and alpha-1-antitrypsin] were all overexpressed in chemoresistant tumors [34], strengthening their potential use as biomarkers.

In an iTRAQ^® ^analysis of serum proteins in patients following brain injury, Hergenroeder et al. [44] identified 160 proteins (≥ 95% confidence) in sera immunodepleted by the IgY12 column. Fifteen of the proteins identified were increased in abundance compared to controls. The relatively small number of serum proteins that they found to be upregulated by iTRAQ^® ^parallels the numbers seen in our study. The proteins that were elevated in serum from patients with traumatic brain injury included many of the same serum proteins we found elevated in ovarian cancer (e.g. haptoglobin, orosomucoids-1 and -2, alpha-1 antitrypsin, serum amyloid P-component, and alpha-1 antichymotrypsin), which suggests that these proteins are not specific to ovarian cancer [44]. However, several of these "acute phase" proteins have recently been shown to discriminate between malignant and benign gynecological disease, and thus may have a role in a "multi-analyte" panel of ovarian cancer biomarkers [45]. In general, the results indicate that the continued presence of highly abundant serum proteins post-depletion, as well as the presence of moderately abundant serum proteins, may interfere with the detection of low abundance proteins that are more specific to the disease.

Recently, Lin et al. [13] reported the identification of serum biomarkers for ovarian cancer using depletion of both high and moderately abundant proteins in combination with LC-MS/MS and ICAT^® ^labeling. Using their "deep depletion" method, they were able to identify 222 proteins, about three times as many as they found with depletion of only the most abundant proteins. Similar to our study, they found LRG1 levels were increased in ovarian cancer serum. In contrast, they reported elevated levels of a number of proteins (e.g., hemopexin, histidine-rich glycoprotein, and vitronectin) that were identified in our study but did not meet our criteria for overexpression in ovarian cancer serum. This is potentially due to the small number of samples (two pools of ten individuals in each group) used in their study.

## Conclusions

Using the iTRAQ^® ^labeling technology, we found an increase in the levels of fourteen proteins in serum from ovarian cancer patients relative to non-cancer controls, demonstrating the utility of this technique for biomarker discovery. In the future, it may be necessary to refine the immunodepletion strategy in order to improve the MS detection of low abundance proteins that are potentially tumor-specific. Tonack et al. [46], recently reported a protocol for iTRAQ^® ^labeling of immunodepleted serum and were able to identify 254 proteins, including tissue leakage proteins present at very low abundance [46]. Although we were able to identify several promising candidates, the presence of moderately abundant serum proteins limited our ability to find additional protein biomarkers. The proteins ECM1, LBP1, and PRG4 all warrant further investigation into their specificity as potential biomarkers for ovarian cancer, using an ELISA or other methods suitable for use in whole (undepleted) serum. It will also be important to validate the candidate biomarkers identified using a large population of individual serum samples, and to include samples from women with early stage ovarian cancer, benign gynecological conditions, and other types of solid tumors, as well as healthy controls.

## Methods

### Patient samples

De-identified serum samples from 60 patients with serous ovarian carcinoma and 60 female non-cancer controls were obtained from the Gynecologic Oncology Group (GOG) Tissue Bank. The majority of the ovarian cancer serum samples were from patients with stage III serous tumors (44 samples), nine had stage IV, and seven had stage I and II tumors. The age of ovarian cancer patients ranged from 35-85 years compared to 19-58 years for the non-cancer controls. Cancer and non-cancer control sera were separately pooled into six random groups, each containing serum from ten patients (C1 to C6 for the 60 cancer samples; N1 to N6 for the 60 non-cancer control samples) as previously described [10].

### Serum depletion

Serum pools were depleted of the six or twelve most highly abundant serum proteins using one of three commercially available immunoaffinity columns: the multiple affinity removal system (MARS) spin column (Agilent Technologies, Santa Clara, CA), the ProteomeLab IgY12 spin column (Beckman Coulter, Fullerton, CA) and the ProteomeLab IgY12 High Capacity LC10 affinity column (Beckman Coulter). Columns were run according to the manufacturer's instructions [10].

### iTRAQ^® ^labeling

Peptides from 100 μg of each sample were labeled with 4-plex iTRAQ^® ^reagents according to the manufacturer's procedure (Applied Biosystems Inc., Foster City, CA). For the medium and low abundance proteins recovered from the MARS column, one pool of ovarian cancer sera (C1) was labeled with isobaric tag 115, while one pool of non-cancer sera (N1) was divided into aliquots that were separately labeled with isobaric tags 116 and 117 (Table 1). Four sets of four samples (two ovarian cancer and two non-cancer serum pools, depleted of high abundance proteins by the IgY12 spin or IgY12 HPLC) were labeled with the iTRAQ^® ^isobaric reporter tags, 114, 115, 116, or 117. Labeled peptides from four samples were combined into one tube and dried *in vacuo*. A SepPac™ C18 cartridge (Waters Corporation, Milford, MA) was used to exchange the buffer, and to remove trypsin and the hydrolyzed unbound iTRAQ^® ^reagents from the labeled peptides.

### Strong cation exchange LC

Strong cation exchange (SCX) liquid chromatography was used to separate the labeled peptides in the first dimension as previously described [47]. Fractions were collected at 3-min intervals and dried *in vacuo*.

### Reversed phase LC-MS/MS analysis

Each dried SCX fraction with an A280 > 2 (fractions 13-23) was reconstituted and injected onto a Dionex/LCP Packings (Sunnyvale, CA) capillary LC system online with a QSTAR Pulsar *i *mass spectrometer (Applied Biosystems) as previously described [47]. Product ion spectra were collected in an information-dependent acquisition (IDA) mode. IDA mode settings included continuous cycles of one full-scan TOF MS from *m/z *400 to 1,100 (1.5 sec) plus four product ion scans from *m/z *50 to 2,000 (3 sec each). Precursor *m/z *values were selected from a peak list automatically generated by Analyst QS software (Applied Biosystems) from the TOF MS scan during acquisition, starting with the most intense ion. The data files have been deposited on the ProteomeCommons.org Tranche network using the following hash: yWlRCpE/tTaPaNyJWqYVXFNGWs/H+29jMKH+aREtnW9HV/fixhy94y6WSlkT+s1ue6ictzVQF7oP5PVtavlddRSir3sAAAAAAAA92w==.

### Data analysis and interpretation

ProteinPilot™ 2.0 software (Applied Biosystems), which employs the Paragon™ search algorithm [48], was used for peptide matching, protein identification, and relative protein quantitation. MS/MS data were searched against the NCBI non-redundant human database (121306) plus common contaminants (179 proteins, Thermo Scientific), for a total of 289,431 proteins. Rates of false positive identifications were estimated using a concatenated reversed sequence database [49]. False positive rates were < 0.3% for all experiments. The search parameters were 95% confidence for the detected protein threshold, trypsin digestion, modification of cysteines by methylmethanethiosulfonate, biological modifications and thorough search effort. The relative abundance of each peptide was determined by ProteinPilot™ using the peak areas of signature ions from the iTRAQ^®^-labeled peptides. The Progroup™ Algorithm within the ProteinPilot™ v2.0 software was used to compile the peptide identification into protein groups and to show protein-based ratios of relative abundance. Peptides shared among related but distinct proteins were not used for quantitation. Peptide MS/MS for protein identifications inferred from single peptide hits were manually inspected. In all cases except two, protein identifications made from single peptide hits in one depletion experiment were made with two or more peptides in at least one of the alternative depletion experiments. Labeled spectra for these two proteins are included as Additional file 3, Figure S1. Proteins were considered overexpressed in ovarian cancer relative to non-cancer control sera if the iTRAQ^® ^ratio of C/N was >1.6 and if the corresponding N/N ratio was between 0.8 and 1.2 in at least one experiment.

### Western Blots

Fifty μg of IgY12 HPLC column depleted sera was separated by SDS-PAGE and transferred to three replicate PVDF membranes as described [10]. The blots were probed with antibodies against human extracellular matrix protein 1 (11521-1-AP, ProteinTech Group, Chicago, IL); lipopolysaccharide binding protein-1 (AF870, R&D Systems, Minneapolis, MN); alpha-1 antitrypsin (mAb G11, Abcam, Cambridge, MA), and detected with a horseradish peroxidase conjugated secondary antibody and chemiluminescence (SuperSignal West Femto Maximum Sensitivity substrate; Pierce, Rockford, IL). Images were collected by exposure to Kodak ×500 film (Midwest Scientific, Valley Park, MO). A replicate PVDF blot was stained for total protein with colloidal gold (BioRad, Hercules, CA) as a loading control.

## Abbreviations

C: cancer; N: non-cancer; MARS: multiple affinity removal system; SCX: strong cation exchange; IDA: information-dependent acquisition; LRG1: leucine-rich alpha-2 glycoprotein-1; PRG4: proteoglycan-4; ECM1: extracellular matrix protein-1; LBP1: lipopolysaccharide binding protein-1; ApoE: apolipoprotein-E.

## Competing interests

The authors declare that they have no competing interests.

## Authors' contributions

KB carried out the Western blot studies, participated in the data analysis and drafted the manuscript. JA contributed to the Western blots, performed the immunodepletion, and participated in the design of the study and data analysis. LA performed the immunodepletion and iTRAQ^® ^labeling, and participated in the design of the study and data analysis. LH participated in the design of the study and data analysis. AS conceived of the study, participated in its design and coordination and helped to draft the manuscript. All authors read and approved the final manuscript.

## Supplementary Material

Additional file 1 **Table S1. Serum Proteins Upregulated by iTRAQ^®^**. Click here for file

Additional file 2 **Table S2. Alphabetized listing of the number of peptides identified per protein**. Click here for file

Additional file 3 **Figure S1. Tandem mass spectra for the single peptide identifications from iTRAQ^® ^experiments**. Spectra for single peptides used to identify C-reactive protein and LBP1 (two experiments) are displayed in Analyst^® ^QS 1.1 software with Bioanalyst Extensions. The amino acid sequence for each peptide is displayed above the spectrum. The b- and y-type ions found are written above the peaks in the spectrum. Click here for file
