# Supplementary figures and images for: Quantitative proteomic analysis by iTRAQ® for the identification of candidate biomarkers in ovarian cancer serum
Source: Proteome Sci. 2010 Jun 14;8:31. doi: 10.1186/1477-5956-8-31 (PMC2893134; doi:10.1186/1477-5956-8-31)

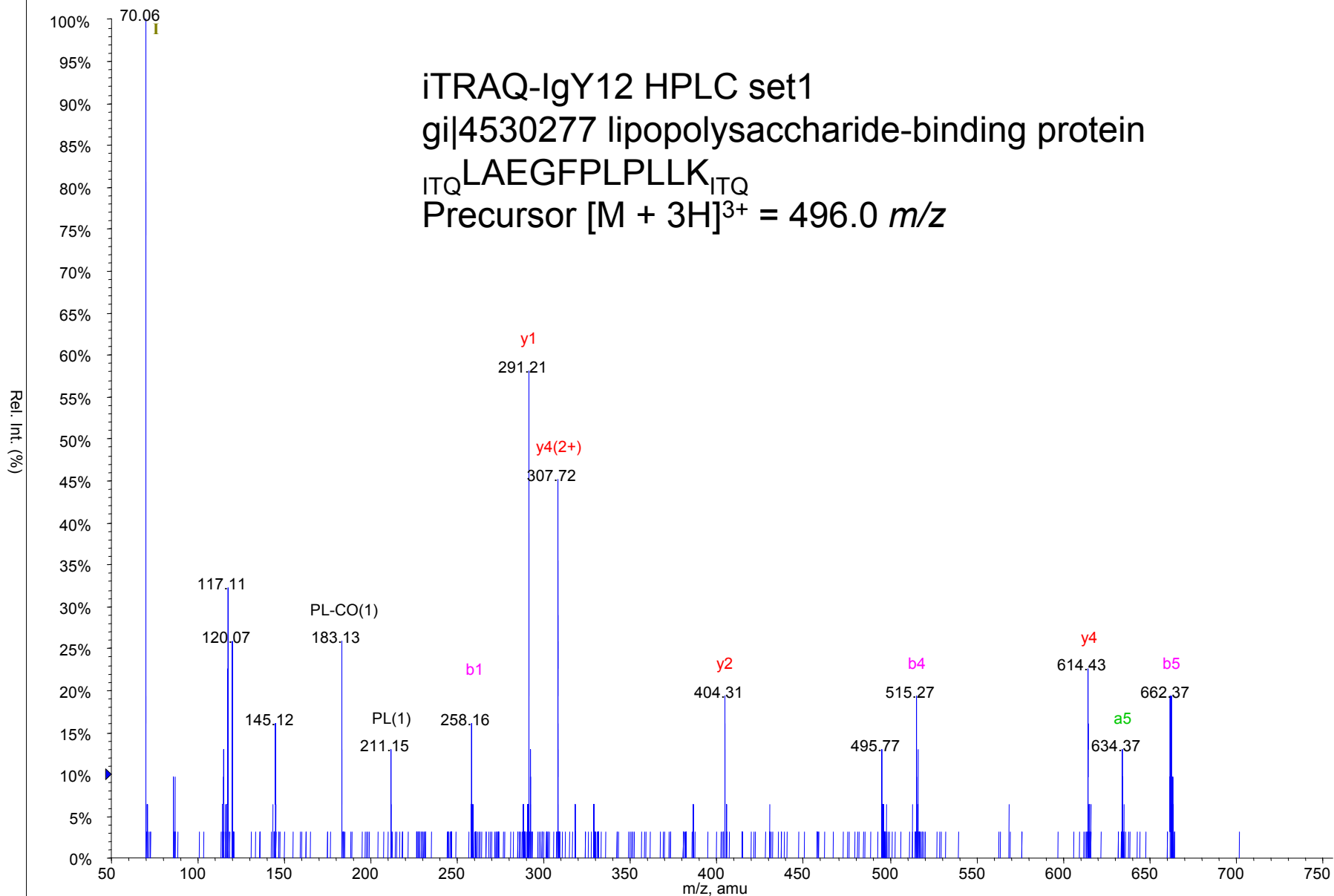

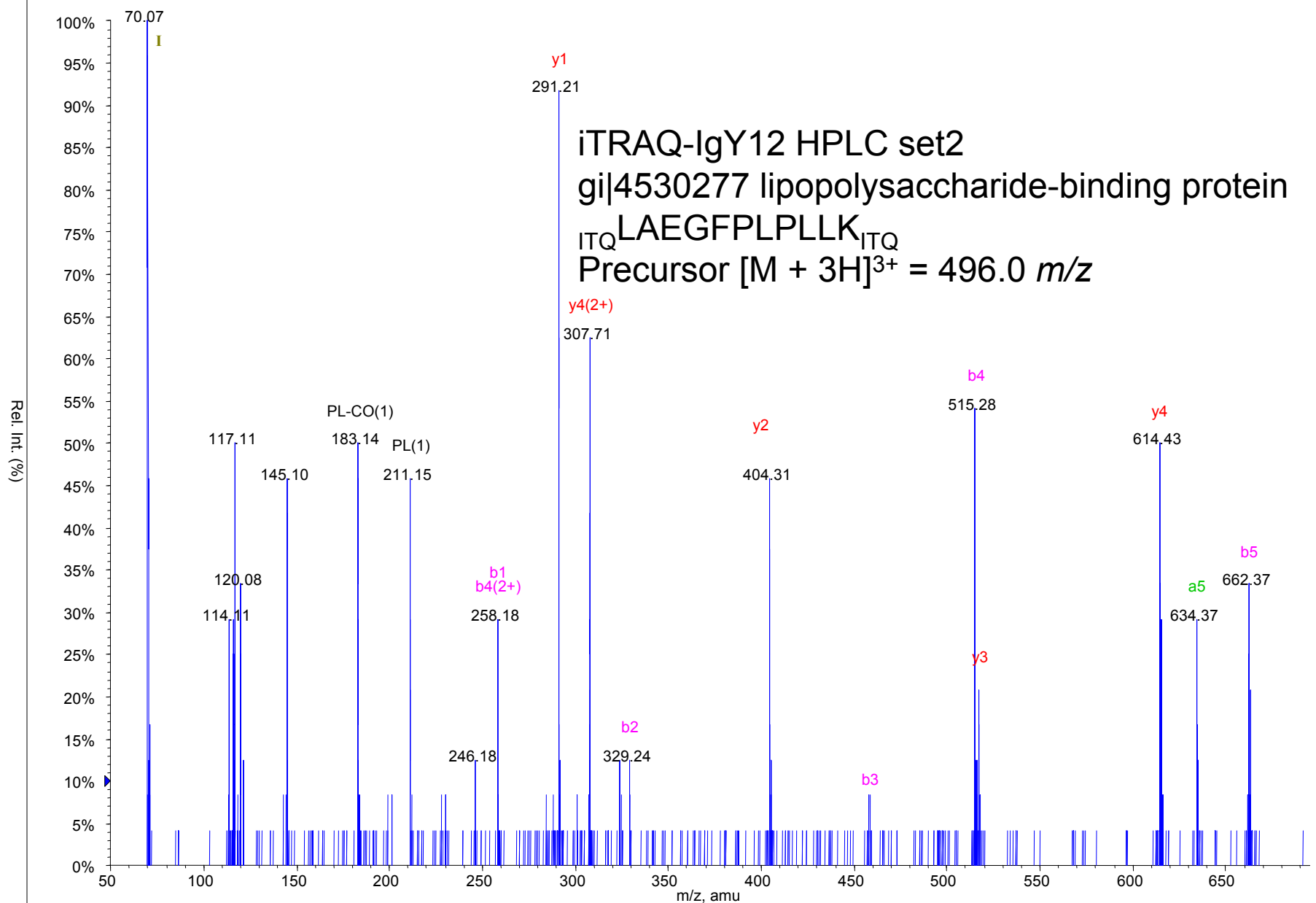

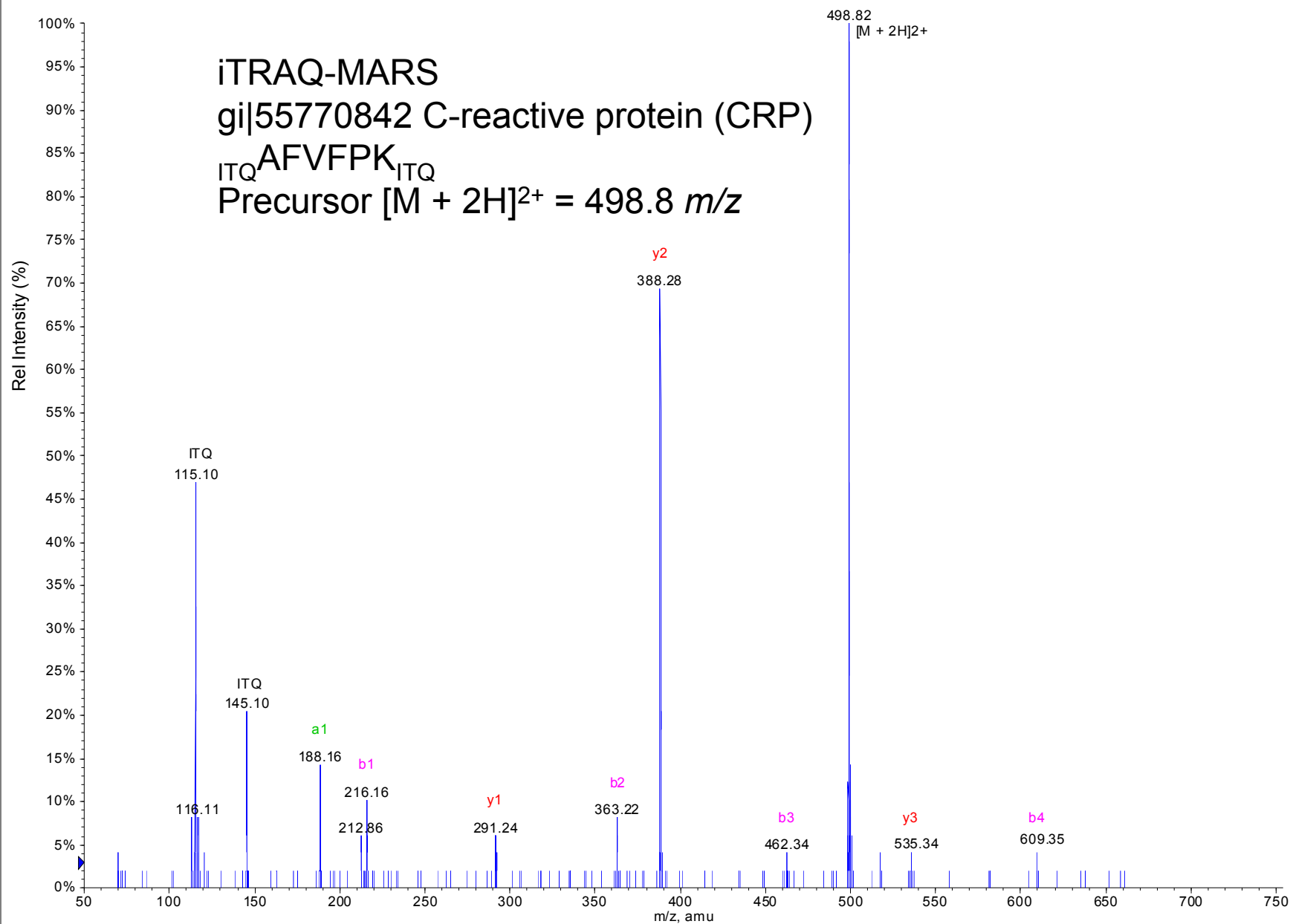

Supplement: Additional file 3 — Figure S1. Tandem mass spectra for the single peptide identifications from iTRAQ® experiments. Spectra for single peptides used to identify C-reactive protein and LBP1 (two experiments) are displayed in Analyst® QS 1.1 software with Bioanalyst Extensions. The amino acid sequence for each peptide is displayed above the spectrum. The b- and y-type ions found are written above the peaks in the spectrum. [file 1477-5956-8-31-S3.PDF]
